# Assessment of validity and comparison of two Spanish versions of the Geriatric Depression Scale

**DOI:** 10.3389/fpsyg.2023.1101886

**Published:** 2023-05-17

**Authors:** Cesar Bugallo-Carrera, Carlos Dosil-Díaz, Arturo X. Pereiro, Luís Anido-Rifón, Moisés Pacheco-Lorenzo, Manuel J. Fernández-Iglesias, Manuel Gandoy-Crego

**Affiliations:** ^1^Department of Developmental Psychology, University of Santiago de Compostela, Santiago de Compostela, Spain; ^2^AtlanTTic Research Center, University of Vigo, Vigo, Spain; ^3^Department of Psychiatry, Radiology, Public Health, Nursing and Medicine, University of Santiago de Compostela, Santiago de Compostela, Spain

**Keywords:** GDS, depression, older adults, validation, Spain

## Abstract

**Introduction:**

The Geriatric Depression Scale is an instrument used to identify depression in people of an older age. The original English version of this scale has been translated into Spanish (GDS-*VE*); two shorter versions of 5- (GDS-5) and 15-items (GDS-15) have been developed.

**Aim of the study:**

To assess the validity and compare the 5- and 15-item Spanish versions of the GDS among the Spanish population.

**Materials and methods:**

573 Galicia residents aged >50 years participated in this study. The following instruments were applied: the 19-item Control, Autonomy, Self-Realization and Pleasure scale, the Subjective Memory Complaints Questionnaire, the Mini-Mental State Examination test, the GDS-5, and the GDS-15.

**Results:**

We found differences in total score between GDS-5 and GDS-15 regarding the variable sex. Internal reliability for GDS-5 and GDS-15 was 0.495 and 0.715, respectively. Sensitivity and specificity for GDS-5 – with a cut-off value of 1 – was 0.517 and 0.650, respectively; for GDS-15 – with a cut-off value of 3 points – sensitivity was 0.755 and specificity 0.668. GDS-5 has a ROC curve of 0.617 and GDS-15 of 0.764.

**Conclusion:**

GDS-15, and to a greater extent GDS-5, should be revised or even reformulated to improve their diagnostic usefulness by choosing higher discriminative ability items or even include new items with greater sensitivity that consider currently prevailing psychosocial factors.

## Introduction

To date, there are over 1,000 million people aged ≥60 years worldwide, with an estimated of 1,400 million by 2030 (WHO, 2022). This increase of people of an older age – associated to longer life expectancy – implies physical and psychological challenges, changes often considered part of the aging process (Blazer and Hybels, 2005). Mental disorders are expected to increase significantly, depression included (Flood and Buckwalter, 2009). A large number of studies from various countries report on the prevalence of depression in people of an older age, although mostly, their data are distinct: from 7.6% in Malaysia to 81.1% in India (Zenebe et al., 2021). This large variability results from the differences between the studied territories regarding the perception/stigma of mental health disorders, the health system, or the instruments used for their detection (Ferrari et al., 2013; Krishnamoorthy et al., 2020; Moreno-Agostino et al., 2021), often leading to a wrong diagnosis (Stek et al., 2004). In older adults, depression is a major public health problem that increases emotional distress and worsens comorbid conditions; consequently, there is a rise in healthcare costs (Luppa et al., 2008), besides a significant increase in suicide and other cause mortality (Aziz and Steffens, 2013; Malhi and Mann, 2018; Jiang et al., 2020).

However, in old age not everything is negative, since it is known that there are a series of differential characteristics of the emotional experience of older people that help explain the phenomenon of the well-being paradox in old age, according to which, Despite the increase associated with old age in the frequency of negative life events such as illnesses or emotional losses, older people continue to maintain similar or higher levels of subjective well-being. These characteristics can be summarized as less negative emotional experience, greater selectivity of emotional information, especially if it is rewarding, and greater control of emotions compared to younger people, presenting greater emotional stability and selecting more situations. In which they get involved, in order to optimize their emotional well-being (Carstensen, 1993; Márquez-González et al., 2004).

Validated instruments are key to identify people with depression – or who are at risk – and act appropriately. Many instruments have been developed to identify depression in older adults, most validated at different times and with diverse populations (Gokcekuyu et al., 2022). Thus, the development of these instruments are influenced by psychosocial factors strongly associated to the period and social context of their validation. This reinforces the interest of having reliable and valid tools to detect depression in people of an older age considering the existing time- and space-related psychosocial risks predominant at the assessment (Areán and Reynolds, 2005).

In 1983, the 30-item Geriatric Depression Scale (GDS-30) was developed (Brink et al., 1982); later, a reduced 15-ited version was created (GDS-15) (Sheikh and Yesavage, 1986). It was proved that these 15- or 30-item tests were difficult to complete for people of an older age with some type of disease and/or limitation due to visual of hearing impairments (Gokcekuyu et al., 2022). Similarly, the quality and length of the exam may be affected in patients with conditions such as chronic pain or dementia. Thus, shorter tests that maintain adequate levels of reliability and validity were needed. Thus, in 1997, Hoyl and collaborators developed the 5-item Geriatric Depression Scale (GDS-5). In Spain, there are several Spanish versions of the GDS validated in community populations, e.g., GDS-*VE*30 (Spanish 30-item Geriatric Depression Scale) (Izal et al., 2007), GDS-15 (Spanish Geriatric 15-item Depression Scale) (de Dios del Valle et al., 2001; Martínez de la Iglesia et al., 2002; Izal et al., 2007; Ortega Orcos et al., 2007), or the GDS-5 (Spanish 5-item Geriatric Depression Scale) (de Dios del Valle et al., 2001; Izal et al., 2007; Ortega Orcos et al., 2007). Moreover, there are validated versions of the GDS-15 and GDS-5 for Spanish populations with cognitive deterioration (Lucas-Carrasco, 2012; Table 1).

**Table 1:** Five- and 15-item versions of the Geriatric Depression Scale and response options.

Are you satisfied with your life?	Yes	No
Have you dropped many of your activities?	Yes	No
Do you feel that your life is empty?	**Yes**	No
Do you often get bored?	**Yes**	No
Are you in good spirits most of the time?	Yes	**No**
Are you afraid that something is going to happen to you?	**Yes**	No
Do you feel happy most of the time?	Yes	**No**
Do you often feel abandoned?	**Yes**	No
Do you prefer to stay home rather than going out?	**Yes**	No
Do you feel you have more problems with memory than most people?	**Yes**	No
Do you think it is wonderful to be alive?	Yes	**No**
Is it hard for you to get started with new projects?	**Yes**	No
Do you feel full of energy?	Yes	**No**
Do you feel your situation is hopeless?	**Yes**	No
Do you feel most people are better off than you are?	**Yes**	No

Questions in bold correspond to the 5-item version of the scale. Responses indicating depression are in bold. Each of these answers counts one point.

Own elaboration.

The purpose of this study was to validate and compare the GDS-5 and GDS-15 scales for the Spanish population and assess their psychometric properties.

## Methods

573 people aged ≥50 years were included in this study (*M* = 70.09; SD = 9.52). All were residents of Galicia (Northwest Spain) and were recruited through socio-cultural, professional, and civic associations. Participants were selected by convenience sampling to obtain a sample equally distributed based on age group, level of education, and sex. Inclusion criteria: aged ≥50 years and without psychiatric diseases or incapacitating sensory or motor deficit. Exclusion criteria: absence of cognitive impairment and illiteracy (participates had to at least know how to read and write). Signed informed consent was obtained from all participants.

Evaluations were performed by psychologists in the homes of the participates and socio-cultural centers. The instruments were applied in the following order, counterbalancing the GDS-5 and GDS-15 scales (Martínez et al., 1993): a sociodemographic questionnaire (Pereiro et al., 2017), the Spanish version of the Mini-Mental State Examination (MMSE, Lobo et al., 1979), the GDS-5 scale (Martínez et al., 1993), the Spanish version of the 19-item Control, Autonomy, Self-realization, Pleasure scale (CASP-19, Netuveli et al., 2006), the Spanish version of the Subjective Memory Complaint Questionnaire (SMCQ, Benedet et al., 1996), and the GDS-15 scales (Martínez et al., 1993).

### Data analysis

The same analyses of data were simultaneously done for both GDS-5 and GDS-15 versions.

Descriptive statistics were obtained from each sociodemographic variables included in the study. Student’s *t*-test for independent samples was applied to determine the difference of total scale score for the variable sex. The relationships between total score for both scales and the variables age and education were assessed using Pearson’s bivariate correlation. To determine which variables significantly and independently contributed on the overall scoring in both scales, different multiple linear regression analysis were done (using the stepwise procedure), including sex, age, years of schooling, and diagnosis of depression as predictive or independent variables. Using this type of analysis, we examined the effect of each independent variable on the total score for GDS-5 and GDS-15.

We used the Kuder–Richardson Formula 20 (KR-20) to assess the internal reliability of the instruments. To validate the constructor, we examined the convergent validity, assessing the correlation between the GDS-5 / GDS-15 scales and the CASP-19 scale, which measures quality of life; similarly, we examined the discriminant validity by correlating the outcomes of the GDS-5/GDS-15 scales with total Subjective Memory Complaints Questionnaire (SMCQ) scores. In both cases, we calculated Spearman’s rank correlation coefficient.

For the criterion or practice validity, we calculated the sensitivity, specificity, and cut-off values for both scales using the Younden index. The informed clinical diagnosis in the sociodemographic questionnaire was used as the gold standard. We analyzed the discriminative capacity of both study scales to differentiate between healthy and depressed subjects using the area under the Receiver Operating Characteristic (ROC) curve.

In order to know the distribution of the items that make up the GDS-15 and GDS-5 scales, the descriptive statistics of each one were obtained: the mean, the standard deviation, the skewness and the kurtosis.

To determine the dimensionality of the questionnaires, a factorial analysis of both was performed. For this, the Kaiser-Meyer-Olkin (KMO) statistic and Bartlett’s sphericity test were used. With a KMO >0.5 and a significant Bartlett’s test, it would prove the existence of an underlying factor structure. The principal component extraction method was used, with Varimax rotation. The criterion of significant factor loadings was used those greater than ≥0.40.

The ceiling and floor effects of the total scores of both scales were analyzed according to the percentage of participants with the lowest (floor) and highest (ceiling) total scores on the scales. Ceiling and floor effects are considered to be present if more than 15% of respondents achieve the highest or lowest possible total score (Terwee et al., 2007). Data were analyzed using SPSS^®^ Statistics 21 for Windows (IBM^®^, Armonk, NY).

## Results

Table 2 shows the descriptive statistics of the sociodemographic variables included in this study. Student’s *t*-test showed differences in total scores for the variable sex for GDS-5 (*t* = −2.399; gl: 567; *p* < 0.05) and GDS-15 (*t* = −4.767; gl: 567; *p* < 0.05). Significant correlations were found between total GDS-5 and GDS-15 scores and the variables age and years of schooling (Table 3). Specifically, a significant positive correlation was seen for both study scales for age and a significant negative correlation for years of schooling.

**Table 2:** Descriptive statistics for the sociodemographic variables.

Variables	*N*	%
Sex (female)	356	62.6
Age		
50–59 years	76	13.4
60–69 years	197	34.6
70–79 years	179	31.5
+ 80 years	117	20.6
Education (years)		
0–4 years	74	13.0
5–8 years	257	45.2
9–13 years	137	24.1
+ 13 years	101	17.8
Diagnosis of depression	58	10.2

Own elaboration.

**Table 3:** Correlations between total GDS-VE5 and GDS-VE15 scores and the variables age and years of schooling.

		GDS-15	GDS-5
Age	Pearson’s correlation	0.157**	0.155**
	Sig. (bilateral)	0	0
	N	569	569
Education	Pearson’s correlation	−0.184**	−0.198**
	Sig. (bilateral)	0	0.064
	N	569	569

GDS-VE, Geriatric Depression Scale (Spanish version).

**p < 0.001.

Own elaboration.

We carried out regression analyses to assess the individual effects of independent variables on GDS-5 and GDS-15 total scores (Table 4). The independent variables years of schooling, being diagnosed with depression, and age are predictors for total GDS-5 score, while the independent variables sex, years of schooling, and being diagnosed with depression are predictors for total GDS-15 score.

**Table 4:** Significance of linear regression analyses of independent variables on total GDS-VE5 and GDS-VE15 scores.

Factors	*B*	Beta	*T*	Sig.
Regression model on GDS-15 score				
Sex	0.752	0.147	3.565	0
Years of schooling	−0.083	−0.118	−2.653	0.008
Diagnosis of depression	−1.305	−0.159	−3.911	0
Age	0.028	0.109	2.468	0.014
Regression model on GDS-5 score				
Years of schooling	−0.051	−0.16	−3.576	0
Diagnosis of depression	−0.501	−0.135	−3.309	0.001
Age	0.011	0.093	2.068	0.039

GDS-VE, Geriatric Depression Scale (Spanish version).

*p* < 0.001.

Own elaboration.

Internal reliability – calculated using KR-20 – was 0.495 for GDS-5 and 0.715 for GDS-15. Convergent validity for GDS-5 and GDS-15 versus CASP-19 scale was *r*_s_ = −0.447 (*p* < 0.001) and de *r*_s_ = −0.566 (*p* < 0.001), respectively, which indicates a significant negative relationship for both study scales; discriminant validity versus SMCQ was *r*_s_ = −0.046 (*p* < 0.001) and de *r*_s_ = 0.048 (*p* < 0.001), respectively, with no significant relationship in any case (Table 5).

**Table 5:** Convergent validity of GDS-VE5 and GDS-VE15 versus the CASP-19 scale and divergent validity versus the SMCQ scale.

		CASP-19	SMCQ
GDS-15	Spearman’s correlation Sig. (bilateral)	−0.566**	0.048
	N	0	0.632
		569	569
GDS-5	Spearman’s correlation Sig. (bilateral)	−0.447**	−0.046
	N	0	0.646
		569	569

GDS, Geriatric Depression Scale.

CASP-19, 19-item Control, Autonomy, Self-realization, Pleasure scale.

SMCQ, Subjective Memory Complaint Questionnaire.

**p < 0.001.

Own elaboration.

Sensitivity and specificity for GDS-5 – with a cut-off point ≥1 – were, respectively, 0.517 and 0.650, while for GDS-15 – with a cut-off point ≥3 – the sensitivity was 0.755 and the specificity 0.668. Regarding discriminative capacity, the ROC curve value for GDS-5 was 0.617 (*p* < 0.001, with 95% confidence interval between 0.544 and 0.689); the ROC curve value for GDS-15 was 0.764 (*p* < 0.001, with 95% confidence interval between 0.691 and 0.837).

Table 6 shows the descriptive statistics of the items that make up the GDS-15 and GDS-5 scales. Thus, a positive skewness can be seen in items 1, 3, 7, 8, 11, and 14 on the GDS-15 scale and in items 1 and 3 of the GDS-5 scale, corresponding to items 1 and 8 of the GDS-15 scale.

**Table 6:** Descriptive statistics of the items that make up the GDS-15 and GDS-5.

Items	Mean	Standard deviation	Skewness	Kurtosis
GDS-15				
1	0.069	0.255	3.385	9.493
2	0.460	0.498	0.158	−1.982
3	0.146	0.354	2.004	2.021
4	0.294	0.456	0.902	−1.191
5	0.226	0.419	1.308	−0.291
6	0.406	0.491	0.381	−1.961
7	0.153	0.360	1.927	1718
8	0.146	0.354	2.004	2.021
9	0.404	0.491	0.389	−1.856
10	0.272	0.445	1.026	−0.951
11	0.029	0.169	5.553	28.941
12	0.342	0.474	0.668	−1.560
13	0.314	0.464	0.803	−1.360
14	0.083	0.277	3.013	7.101
15	0.232	0.422	1.272	−0.382
GDS-5				
1	0.071	0.257	3.333	9.143
2	0.298	0.457	0.883	−1.224
3	0.150	0.357	1.965	1.866
4	0.399	0.490	0.411	−1.838
5	0.345	0.475	0.651	−1.581

Own elaboration.

The KMO statistic was 0.740 on the GDS-15 scale and 0.590 on the GDS-5 scale, while the Bartlett sphericity test was significant both on the GDS-15 scale (*χ*^2^ = 863.273; gl = 105; *p* < 0.001) and the GDS-5 scale (*χ*^2^ = 75.697; gl = 10; *p* < 0.001), indicating an underlying structure in both.

A first analysis, following the “eigenvalue greater than 1,” showed in the GDS-15 scale a structure of five factors that explained 51.04% of the variance. A first factor was defined that groups items 1 and 3, a second factor that groups items 6, 4, 14, 8, 2, and 9, a third factor that groups items 13 and 5, a fourth factor that groups items 10, 12, and 15, and a fifth factor that groups items 11 and 7. A second analysis showed a structure of 2 factors on the GDS-5 scale that explained 49.65% of the variance: a first factor that groups items 1 and 3 (items 1 and 8 of the GDS-15 scale) and a second factor that groups items 2, 4 and 5 (items 4, 9 and 12 of the GDS-15 scale). All items presented a load greater than 0.40, with the exception of items 2 and 9 on the GDS-15 scale.

Regarding the ceiling effect, this has not been evidenced in any of the scales, however, the floor effect has been present in the GDS-5 scale with 28.6%.

## Discussion

We wanted an instrument to identify depression in people of an older age that would take into account psychometric properties. In this study, we aimed to validate and compare the 5- and 15- item GDSs in the Spanish population, analyzing their psychometric properties.

Regarding validation of the GDS-5 and GDS-15, different total scores are obtained for the variable sex, a significant positive relationship for the variable age, and a significant negative relationship for the variable years of schooling. Our results are in line with most works on the topic, were sex- (de Dios del Valle et al., 2001; Martínez de la Iglesia et al., 2002), age- (De los Santos and Carmona Valdés, 2018), and years of schooling-related (De los Santos and Carmona Valdés, 2018; Gokcekuyu et al., 2022) differences are described. On the variables that act as predictors of total GDS-5 and GDS-15 scores, years of schooling and having a diagnosis of depression are common predictors for both scales. The independent variable age only acts as a predictor of total score for GDS-5 and the independent variable sex only as a predictor of total score for GDS-15.

The 0.715 and 0.495 internal reliability for GDS-15 and GDS-5, respectively, indicates that the fewer the number of items in the scale lesser the internal reliability. Our values are similar to the work by de Dios del Valle et al. (2001), who reported an internal reliability of 0.79 and 0.45 for GDS-15 and GDS-5, respectively, far from the 0.99 found by Martínez de la Iglesia et al. (2002) for GDS-15. The abovementioned studies were done on Spanish populations. The high internal reliability reported by Martínez de la Iglesia et al. (2002) may imply the existence of superfluous items that disregard relevant information about the features to be measured (Barrios and Cosculluela, 2013).

GDS-15 is suitable as a screening instrument to detect the possible presence of depression in people of an older age with adequate sensitivity (78.5%) and acceptable specificity (66.8%) for a cut-off value of 3. GDS-5 has a relatively low sensitivity and acceptable specificity, contrary to what has been reported elsewhere (de Dios del Valle et al., 2001; Ortega Orcos et al., 2007). The diagnostic capacity of the scale significantly worsens when the length of the scale is reduced, same as reported by Izal et al. (2007).

The 5-factor structure found in the GDS-15 scale is different from the two-factor structure found by Lucas-Carrasco (2012) in cognitively healthy subjects and more similar to the four-factor structure found by Weintraub et al. (2007) in subjects with mild dementia. Undoubtedly, this is a topic that should be studied in depth in order to obtain an instrument with a well-defined factorial structure that leads to significant clinical utility in the future.

There are some limitations to this study. On the one hand, the diagnosis of depression was accepted as such only considering the self-report of the evaluated person. There was no rigorous evaluation of study participants by a member of our research team to confirm the possible diagnosis of depression or identify depression in subjects without a clear diagnosis, or who hid their condition. On the other hand, another limitation is the possible existence of a cohort effect. Here, we tried to overcome the possible existence of this effect – detected in previous studies – aiming to validate the scales for the Spanish population (de Dios del Valle et al., 2001; Martínez de la Iglesia et al., 2002; Izal et al., 2007; Ortega Orcos et al., 2007). However, while we expect the possible overcoming, our study may inherently carry the same cohort effect, which in turn will have to be overcome in further validations with a different cohort.

## Conclusion

The evidence in this work indicates that GDS-15, and to a greater extent GDS-5, should be revised or even reformulated to improve their diagnostic usefulness by choosing higher discriminative ability items or even include new items with greater sensitivity that consider currently prevailing psychosocial factors.

Likewise, it must be made clear that the usefulness of the scales is merely to screen for the existence of a possible depressive syndrome, since the confirmation of the diagnosis is exclusively clinical, through an interview conducted by a trained professional.

## Data availability statement

The raw data supporting the conclusions of this article will be made available by the authors, without undue reservation.

## Ethics statement

Ethical review and approval were not required for the study of human participants in accordance with the local legislation and institutional requirements. Written informed consent was obtained from all participants.

## Author contributions

CB-C and CD-D: conception or design of the work. AP: data collection. MP-L, MF-I, and LA-R: data analysis and interpretation. LA-R: drafting the article. MG-C: critical revision of the article. CD-D: final approval of the version to be publish. All authors contributed to the article and approved the submitted version.

## Funding

This work was supported by Grant PID2020-115137RB-I00 funded by MCIN/AEI/10.13039/501100011033.

## Conflict of interest

The authors declare that the research was conducted in the absence of any commercial or financial relationships that could be construed as a potential conflict of interest.

## Publisher’s note

All claims expressed in this article are solely those of the authors and do not necessarily represent those of their affiliated organizations, or those of the publisher, the editors and the reviewers. Any product that may be evaluated in this article, or claim that may be made by its manufacturer, is not guaranteed or endorsed by the publisher.
